# Exploration of risk factors and characteristics of COVID-19 infection among patients with hematological malignancies in Suzhou, China: a retrospective study

**DOI:** 10.3389/fonc.2024.1487516

**Published:** 2025-02-21

**Authors:** Yao Yao, Wenjuan Di, Fangkai He, Bin Liu, Xue Chen, Xiaojun Guan, Zhou Huang, Ying Wang, Depei Wu

**Affiliations:** ^1^ National Clinical Research Center for Hematologic Diseases, Jiangsu Institute of Hematology, The First Affiliated Hospital of Soochow University, Suzhou, China; ^2^ Pulmonary and Critical Care Medicine, Kunshan Hospital of Traditional Chinese Medicine, Suzhou, China; ^3^ Department of Hematology, Soochow Hopes Hematonosis Hospital, Suzhou, China; ^4^ Department of Radiology, The Affiliated Suzhou Hospital of Nanjing Medical University, Suzhou Municipal Hospital, Suzhou, China; ^5^ Department of Pulmonary and Critical Care Medicine, The First Affiliated Hospital of Soochow University, Suzhou, China; ^6^ Department of Radiology, The First Affiliated Hospital of Soochow University, Suzhou, China

**Keywords:** hematological malignancy, COVID-19, mortality, indicator, prognosis

## Abstract

**Background and aim:**

Patients diagnosed with cancer, particularly those with hematologic malignancies, frequently exhibit a state of immunosuppression. Currently, there remains a scarcity of dependable biomarkers for assessing the severity of COVID-19 in individuals with hematologic malignancies. We conducted a retrospective study of morbidity and mortality in patients with hematological malignancies (HM) who had contracted COVID-19. The aim was to offer a reference for clinical diagnosis and treatment.

**Methods:**

A total of 71 patients with HM-confirmed COVID-19 were enrolled from December 2022 to May 2023. Clinical symptoms, laboratory findings, and treatment approaches were collected and documented. Patients were classified into survival and death groups based on their COVID-19 outcomes, and statistical analysis was performed on the clinical data from both groups.

**Results:**

Among the 71 patients, 57 (80.3%) were alive, and 14 (19.7%) had died. The mean age of patients in the death group was significantly higher than that of the survival group (51.29 ± 20.76 *vs*. 49.47 ± 13.04, P=0.030). The proportion of patients receiving mechanical ventilation was significantly higher in the death group (P<0.001). The mortality rate was significantly higher in the critically severe group compared to the mild, moderate, and severe groups (P<0.001). Correlation analysis revealed that certain laboratory indicators lactic acid dehydrogenase (LDH), albumin (ALB), creatine kinase (CK), troponin T (TnT), N-terminal pro-brain natriuretic peptide (NT-proBNP) and fibrin degradation product (FDP), which exhibited significant differences between groups, were significantly correlated with COVID-19-related mortality (all P<0.05). The Cox proportional hazards model indicated that LDH was an independent risk factor associated with the prognosis of HM-confirmed COVID-19.

**Conclusion:**

Patients with hematologic malignancies suffer severe morbidity and mortality due to COVID-19 infection. LDH may serve as a risk factor associated with prognosis in the treatment of COVID-19. Monitoring variations in LDH levels can assist healthcare providers in evaluating disease progression, adjusting treatment plans in a timely manner, and predicting patient outcomes.

## 1 Introduction

The COVID-19 pandemic, caused by severe acute respiratory syndrome coronavirus 2 (SARS-CoV-2), emerged and gained global attention in late 2019 (1), swiftly becoming a major global public health concern due to its respiratory transmission (2). The World Health Organization (WHO) has reported 770,437,327 confirmed cases of COVID-19 worldwide, resulting in 6,956,900 fatalities (3). While the WHO no longer classifies it as a public health emergency of international concern, isolated COVID-19 outbreaks persist in the post pandemic era (4).

COVID-19 typically presents mild to moderate symptoms but can be severe or life-threatening for 5-10% of patients, leading to long-term complications (5). Although COVID-19 has a widespread impact on global health, its effects are particularly pronounced in certain patient populations, such as those with hematologic malignancies (HM) (6). Recent research demonstrates that the mortality rate of COVID-19 is significantly higher in patients with hematological malignancies compared to the general population. Consequently, it is essential to devise targeted treatment and prevention strategies for this particular patient demographic (7, 8).

The study examined the clinical baseline features and hematological markers of COVID-19 infection in patients with hematological malignancies to identify significant risk factors associated with a poor prognosis. These findings should be carefully considered in the diagnostic and treatment processes.

## 2 Materials and methods

### 2.1 Design and patients

We conducted a retrospective data collection of hospitalized hematologic malignancy patients who tested positive for COVID-19 through PCR at the hematology department from December 2022 to May 2023. Data on demographic, clinical, therapeutic, and laboratory information for 71 patients with positive viral RNA detection were extracted from the electronic medical records system. Eligible patients met the inclusion criteria of having WHO-defined hematologic malignancies and confirmed symptomatic COVID-19 infection.

### 2.2 Clinical classification of COVID-19 patients

The severity of COVID-19 at admission was graded according to the clinical classification of COVID-19 patients in China (9).

### 2.3 Outcomes

In this study, we assessed whether the causes of death among the subjects were attributable to COVID-19, adhering to the guidelines established by the ICD and WHO.

Death—patients infected with COVID-19 and dying from COVID-19

Survival—patients infected with COVID-19 and final outcome of survival or death not attributable to COVID-19.

### 2.4 Data collection

The indicators recorded were patients’ characteristics (age, sex, length of stay, HM type, HM characteristics, previous anti-cancer treatment, symptoms of COVID-19 infection, COVID-19 clinical classification (9), anti-viral therapy, mechanical ventilation) and laboratory examinations before therapy [white blood cells (WBCs), neutrophils, lymphocytes, eosinophils, neutrophils/lymphocytes (NLRs), hemoglobin, platelets, alanine transaminase (ALT), lactic acid dehydrogenase (LDH), albumin (ALB), creatine (CR), creatine kinase (CK), troponin T (TnT), N-terminal pro-brain natriuretic peptide (NT-proBNP), D-dimer, fibrin degradation product (FDP), and procalcitonin (PCT)] prior to the administration of COVID-19 treatment. The diagnosis of HM was made according to the WHO classification of hematopoietic tumors (10).

### 2.5 Statistical analysis

Continuous variables that conformed to a normal distribution were expressed as mean ± standard deviation, and comparisons between groups were made using the independent samples t test; continuous variables that did not conform to a normal distribution were expressed as M (Q1,Q3), and comparisons between groups were made using the Mann−Whitney U test. Categorical variables were expressed as proportions (%), and comparisons between groups were made using the chi-square test. Spearman’s test was used to test the correlation between the indicators and death. One-way analyses of death-related influencing factors were performed using the log-rank test. A multifactorial Cox proportional risk regression model was used to analyze the influencing factors of death in patients with hematological malignancies. P<0.05 was considered statistically significant. The statistical data was analyzed using IBM SPSS Statistics for Windows (IBM Corp. Version 26.0. Armonk, NY, USA).

## 3 Results

### 3.1 Baseline characteristics of HM patients with COVID-19

The baseline characteristics of patients with hematological malignancies are presented in **Table 1**. Seventy-one patients with HM were diagnosed with COVID-19, consisting of 39 (54.9%) males and 32 (45.1%) females. The mean age of these patients was 49.83 years (SD=14.72), and the mean duration of hospitalization was 24.72 days (SD=15.47). Patients in the group who died were significantly older than those in the survival group (51.29 years *vs* 49.47 years, P=0.030). The distribution of hematological malignancies was as follows: aggressive lymphoma (LM) 32.4%, multiple myeloma (MM) 5.6%, acute leukemia 56.3%, and myelodysplastic syndrome 5.6%. The specific subtypes of lymphoma and leukemia patients are shown in **Supplementary Table 1**. Among the patients, 59.2% achieved MRD-negative complete remission in prior hematological malignancies. Additionally, in their electronic medical records, patients had received anti-cancer treatments, including hematopoietic stem cell transplant (HSCT) (23.9%), chimeric antigen receptor T-cell (CAR-T) therapy (8.5%), anti-CD20 monoclonal antibody therapy (32.4%), and blinatumomab (1.4%). The prevalent symptoms of COVID-19 infection included cough (31.0%), fever (71.8%), and chest distress with shortness of breath (22.5%). Clinical classification of the patients, as per the ninth edition of the COVID-19 guidelines of the People’s Republic of China, included mild (52.1%), moderate (16.9%), severe (18.3%), and critical severe (12.7%). All patients received one or more antiviral treatments, including paxlovid (47.9%), molnupiravir (59.2%), or azvudine (35.2%). Nineteen patients (26.8%) received mechanical ventilation, and 20 patients (28.1%) received anticoagulant therapy. Mortality events significantly differed across clinical classifications, with critical severe patients having a significantly higher mortality rate than the other three groups (χ2 = 46.096, P<0.001). Patients requiring mechanical ventilation exhibited a significantly higher mortality rate than those who did not necessitate noninvasive or invasive ventilation (χ2 = 23.884, P<0.001).

**Table 1:** Clinical indicators in hematological malignancies suffering from COVID-19 survival and death groups [n/ (mean ± standard deviation)].

Clinical Indicators	Survival(n=57)	Death(n=14)	T/χ^2^	P
Sex, n			1.027	0.311
Male	33	6		
Female	24	8		
Age, years (mean (SD))	49.47 ± 13.04	51.29 ± 20.76	-0.312	0.030*
Length of stay, days (mean (SD))	24.93 ± 14.86	23.93 ± 18.28	0.189	0.557
HM, n			4.293	0.231
Lymphoma	17	6		
Multiple myeloma	2	2		
Leukaemia	35	5		
Myelodysplastic syndrome	3	1		
Remission of hematologic malignancies, n			1.917	0.166
Complete remission	36	6		
Active	21	8		
Previous anti-cancer treatment, n			0.550	0.908
HSCT	13	4		
CAR-T therapy	5	1		
Anti-CD20 monoclonal antibody therapy	17	6		
Blinatumomab	1	0		
Clinical classification, n			46.096	<0.001*
Mild	37	0		
Moderate	9	3		
Severe	11	2		
Critical severe	0	9		
Mechanical ventilation, n			23.884	<0.001*
No	49	3		
Yes	8	11		
Anticoagulation therapy, n			0.491	0.484
No	42	9		
Yes	15	5		

SD, standard deviation; HM, hematological malignancy; HSCT, Haematopoietic stem cell transplant; CAR-T, Chimeric antigen receptor T-cell; t-test: Age; Length of stay; χ2 test: Sex; HM; Remission of hematologic malignancies; Previous anti-cancer treatment; Clinical classification; Mechanical ventilation; Anticoagulation therapy; *P<0.05.

### 3.2 Laboratory indicators in HM patients suffering from COVID-19

**Table 2** shows the differences in features of laboratory blood examinations between the survival and groups of HM patients. There were significant differences in several laboratory indicators between the two groups. The results of routine blood tests showed that lymphocytes (LYM) and eosinophils (EOS) were significantly lower in the death group (all P<0.05). In addition, the levels of ALT, LDH, ALB, and CR were significantly different between the two groups of patients. Levels of ALT, LDH, and CR were elevated in the patients in the death group, whereas levels of ALB were decreased. Additionally, myocardial function tests showed that the levels of CK, TnT, and NT-proBNP were significantly elevated in the death group. Significantly higher levels of D-dimer and FDP were observed in the death group patients. PCT levels were significantly higher in the death group.

**Table 2:** Laboratory indicators in HM patients conformed COVID-19 [(mean ± standard deviation)].

Laboratory indicators	Survival group	Death group	T	P
WBC, (10^9/L)	9.07 ± 22.01	7.52 ± 11.25	0.369	0.605
NE, (10^9/L)	3.28 ± 4.33	3.65 ± 3.49	-0.342	0.936
LYM, (10^9/L)	4.09 ± 11.67	0.89 ± 1.07	2.039	0.046*
EOS, (10^9/L)	0.06 ± 0.17	0.01 ± 0.01	2.431	0.018*
NLR	11.88 ± 54.59	15.55 ± 19.08	-0.414	0.972
HB, (g/L)	95.26 ± 22.12	80.07 ± 26.95	1.954	0.341
PLT, (10^9/L)	118.72 ± 82.58	87.71 ± 65.35	1.504	0.557
ALT, (U/L)	28.78 ± 22.50	52.31 ± 40.36	-2.030	0.007*
LDH, (U/L)	299.87 ± 191.46	882.66 ± 853.55	-2.539	0.000*
ALB, (g/L)	36.44 ± 4.58	32.25 ± 2.97	4.196	0.039*
CR, (umol/L)	56.25 ± 20.50	73.64 ± 70.84	-0.909	0.000*
CK, (U/L)	37.41 ± 29.21	1701.92 ± 3660.13	-1.702	0.000*
TnT, (ng/mL)	11.64 ± 8.71	66.22 ± 90.90	-2.243	0.000*
NT-proBNP, (pg/mL)	491.78 ± 1197.43	1884.51 ± 3406.98	-1.500	0.017*
D-dimer, (ug/mL)	1.91 ± 3.46	5.87 ± 6.43	-2.146	0.000*
FDP, (mg/L)	6..21 ± 10.99	16.09 ± 26.35	-1.371	0.004*
PCT, (ng/mL)	0.15 ± 0.18	0.58 ± 1.00	-1.584	0.000*

WBC, White blood cell; NE, Neutrophils; LYM, Lymphocytes; EOS, Eosinophils; NLR, Neutrophils/Lymphocytes; HB, Hemoglobin; PLT, Platelet; ALT, Alanine transaminase; LDH, Lactic acid dehydrogenase; ALB, Albumin; CR, Creatine; CK, Creatine kinase; TnT, Troponin T; NT-proBNP, N-Terminal Pro-Brain Natriuretic Peptide; FDP, Fibrin degradation product; PCT, Procalcitonin; t-test: WBC; LYM; NLR; HB; PLT; ALT; LDH; ALB; CR; CK; TnT; NT-proBNP; FDP; PCT; *P<0.05.

### 3.3 Correlation between indicators and death due to COVID-19

**Figure 1:**
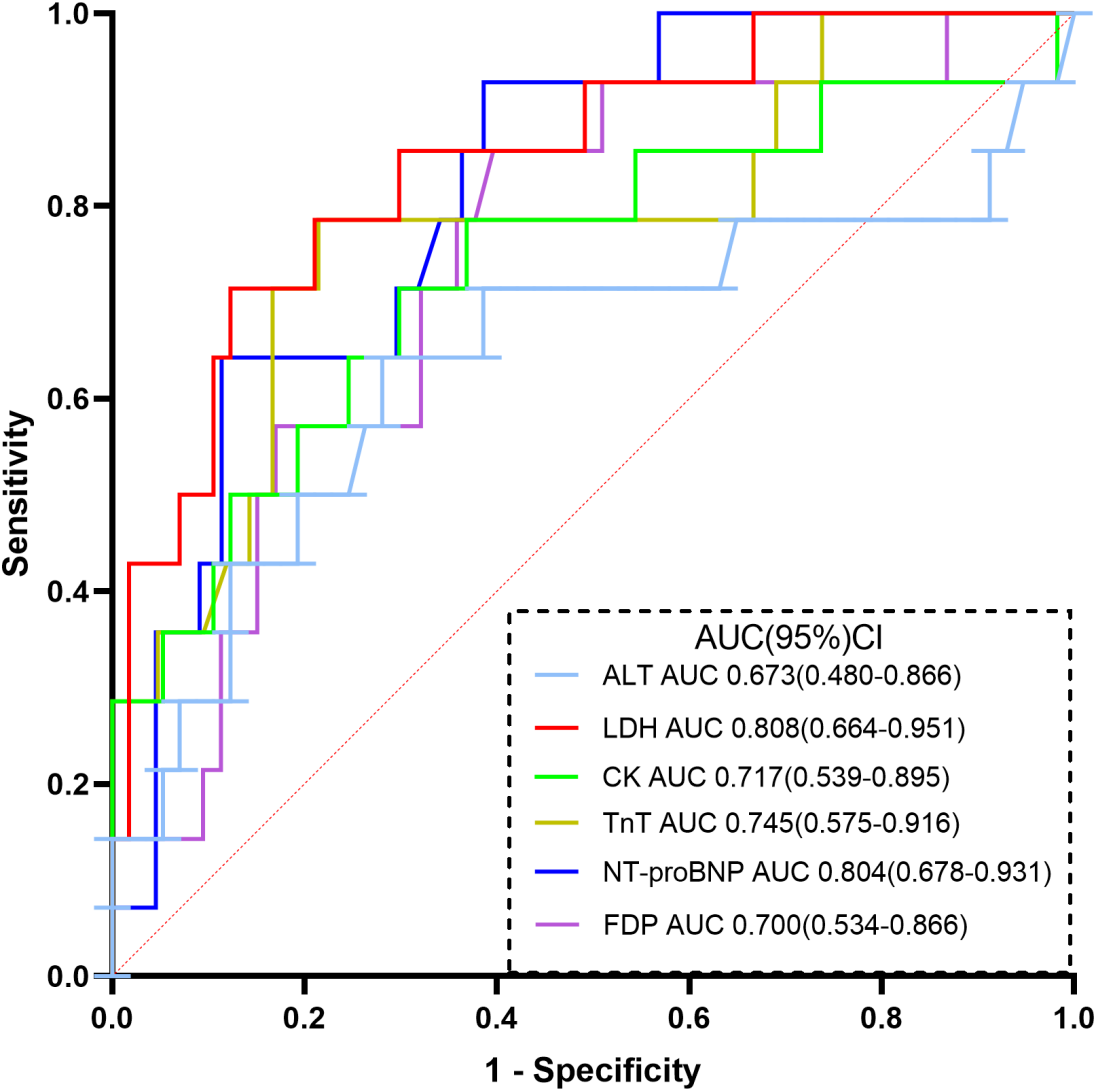
ROC curve analysis of each indicator in the diagnosis of death due to COVID-19.

We included continuous variables with statistically significant differences in clinical and laboratory indicators between the two groups to investigate their correlation with COVID-19-related mortality in patients with hematological malignancies. The results are presented in **Table 3**. Correlation analysis demonstrated that the levels of LYM, EOS and D-dimer were associated with mortality. Additionally, the levels of LDH, ALB, CK, TnT, NT-proBNP, and FDP showed significant associations with mortality. ROC curves were utilized to analyze indicators with strong correlations (**Figure 1**), classifying them into groups above and below a specified threshold. The sensitivity and specificity of these strongly correlated indicators are shown in **Table 4**.

**Table 3:** Correlation between indicators and death due to COVID-19.

Indicators	Death due to COVID-19	
	r	P
Age,years	0.090	0.456
Length of stay,days	-0.103	0.399
LYM, (10^9/L)	-0.247	0.038*
EOS, (10^9/L)	-0.272	0.022*
ALT, (U/L)	0.200	0.094
LDH, (U/L)	0.478	<0.001**
ALB, (g/L)	-0.359	0.002**
CR, (umol/L)	-0.078	0.519
CK, (U/L)	0.330	0.005**
TnT, (ng/mL)	0.411	0.002**
NT-proBNP, (pg/mL)	0.471	<0.001**
D-dimer, (ug/mL)	0.275	0.025*
FDP, (mg/L)	0.346	0.004**
PCT, (ng/mL)	0.188	0.139

LYM, Lymphocytes; EOS, Eosinophils; ALT, Alanine transaminase; LDH, Lactic acid dehydrogenase; ALB, Albumin; CR, Creatine; CK, Creatine kinase; TnT, Troponin T; NT-proBNP, N-TerminalPro-Brain Natriuretic Peptide; FDP, Fibrin degradation product; PCT, Procalcitonin. *P<0.05: There is a correlation between the two indicators, **P<0.01: There is a strong correlation between the two indicators.

**Table 4:** Clinical diagnostic efficacy of 6 strongly correlated indicators.

Indicators	cut-off	Sensitivity	Specificity	AUC	AUC 95%CI	
					lower	upper
ALT, (U/L)	23.15	0.769	0.588	0.673	0.480	0.866
LDH, (U/L)	468.80	0.692	0.853	0.808	0.664	0.951
CK, (U/L)	61.75	0.538	0.824	0.717	0.539	0.895
TnT, (ng/mL)	14.12	0.769	0.765	0.745	0.575	0.916
NT-proBNP, (pg/mL)	202.75	0.923	0.618	0.804	0.678	0.931
FDP, (mg/L)	2.80	0.846	0.559	0.700	0.534	0.866

ALT, Alanine transaminase; LDH, Lactic acid dehydrogenase; CK, Creatine kinase; TnT, Troponin T; NT-proBNP, N-Terminal Pro-Brain Natriuretic Peptide; FDP, Fibrin degradation product; AUC, Area Under Curve.

### 3.4 Cox proportional hazards analysis of prognosis-related indicators

**Figure 2:**
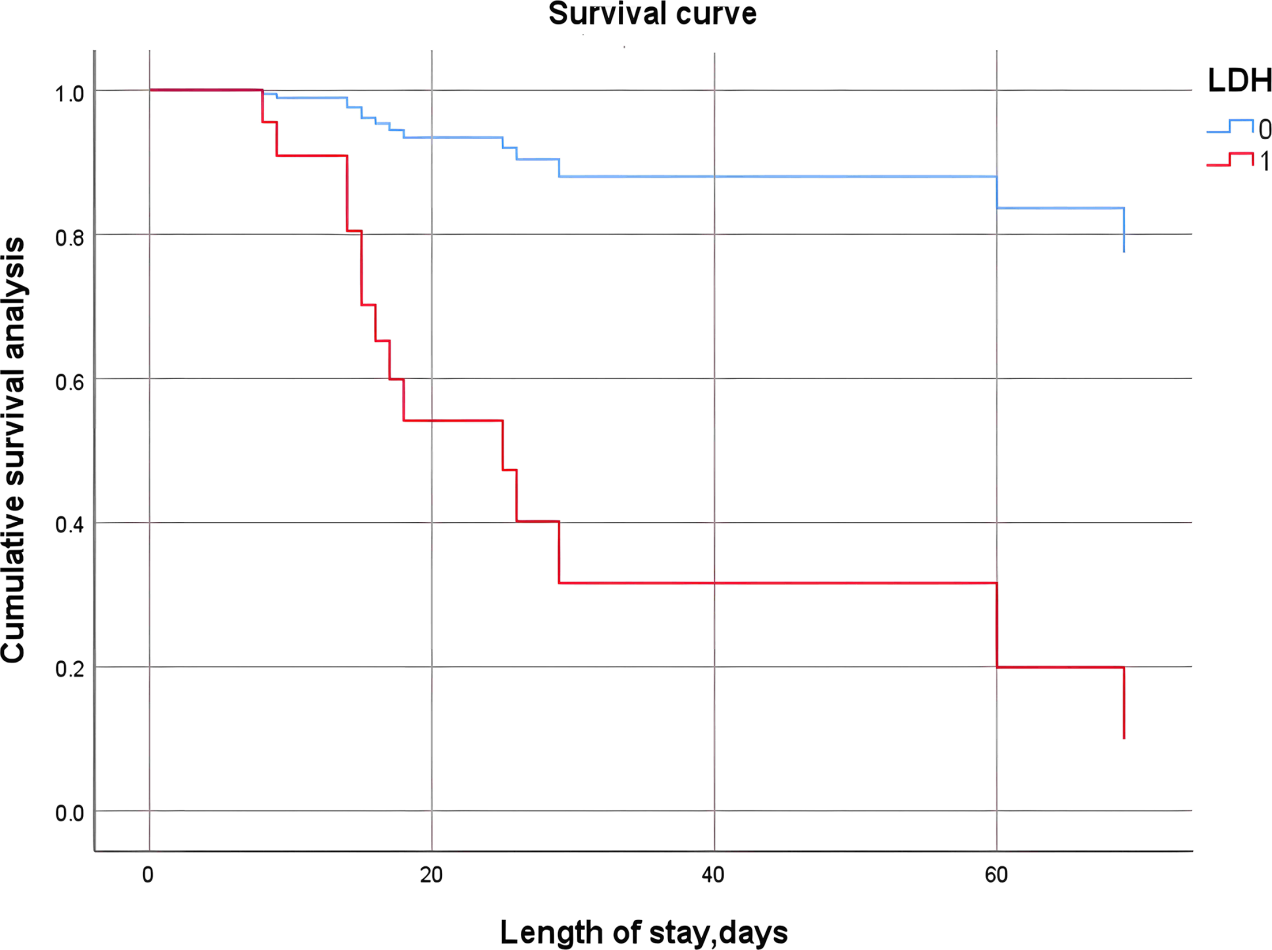
K−M survival curve analysis of the high-level and low-level LDH groups. LDH-0: LDH<468.80 U/L; LDH-1: LDH≥468.80 U/L.

Continuous variables were transformed into categorical variables based on the cutoff values described above. We defined those above the cutoff value as high-level groups and those below the cutoff value as low-level groups. We included categorical variables that were statistically significant in terms of clinical characteristics, such as clinical classification and mechanical ventilation, in the Cox proportional hazards analysis along with the categorical variables after the definition above. The final results showed that only LDH was an independent risk factor associated with prognosis, that is, death. We further plotted survival curves based on LDH and length of stay in the hospital. Kaplan−Meier (K−M) survival curve (**Figure 2**) analysis showed that patients in the high-level LDH group were at higher risk of death (χ2 = 18.021, P<0.001).

## 4 Discussion

Patients with hematological malignancies face a higher risk of severe COVID-19 outcomes due to weakened immune systems, with studies indicating higher mortality compared to the general population (11, 12). Our retrospective study aims to analyze clinical data of hospitalized patients to identify COVID-19 prognosis risk factors in this group, enhancing understanding of disease severity and patient outcomes post-pandemic. Our study found that older patients, those classified as severe, and those needing mechanical ventilation had a higher mortality risk. Similarly, Professor Hosseini-Moghaddam’s report (13) emphasizes the significance of age in relation to adjusted mortality by sex. Their study also found that the mean age of patients with hematological malignancies suffering from COVID-19 exceeded that of patients with solid tumors. Clinical severity correlated with higher mortality, linked to symptoms, pulmonary lesions, and multi-organ involvement in critically ill patients, who also had a higher need for mechanical ventilation.

Earlier studies have found that lymphocyte counts are reduced in most COVID-1 9 patients, and the reduction is more pronounced in the critically ill group (8, 14). A review of COVID-19 by Grifonin A et al. (15) showed that lymphocyte counts, CD4+ T-lymphocyte counts, and CD8+ T-lymphocyte counts were persistently low in the death group. Conversely, in the survival group, lymphocyte counts were higher and gradually returned to normal as the disease improved. It was concluded that COVID-19 virus may act on lymphocytes, especially T lymphocytes, resulting in a decrease in both CD4+ T lymphocytes and CD8+ T lymphocytes, and therefore, damage to T lymphocytes may be an important factor contributing to the deterioration of the patient’s condition. For patients with hematologic malignancies, due to frequent chemotherapy and immunotherapy, the lymphocytes themselves are damaged, which also becomes a susceptibility factor for COVID-19, and HM patients are also prone to a high incidence of critically ill patients. In our study, pretreatment lymphocyte counts were lower in the deceased group than in the surviving group, but results regarding T-cell subsets are lacking.

In this study, we demonstrated that early eosinophil (EOS) counts in COVID-19 patients with hematological malignancies were correlated with higher mortality, and most of the dead patients had EOS counts as low as 0. Therefore, to some extent, EOS counts may serve as an important reference for the evaluation of the disease conditions of patients with COVID-19. Previous studies have simply concluded that eosinophils are mainly involved in the immune process of allergic diseases, such as parasites as inflammatory cells only. In fact, EOS are critically important cells in the process of immune and allergic responses, and some studies have shown that EOS can promote host cell immunity, thereby reducing the replication of influenza virus H1N1 in the lungs and improving the clearance of the virus (16, 17). In addition to influenza virus, decreased EOS counts have also been observed in SARS and other respiratory virus infections (17). Changes in the number of eosinophils are related to the level of the adrenal cortex in the body; when the concentration of glucocorticoids decreases, the number of eosinophils is increased. When the patient suffers from acute viral infection, the adrenocorticotropic hormone will increase stress secretion, which promotes the body’s anti-infection, inhibit the inflammatory response, so in the early stage of eosinophils is reduced, but after entering the clinical intervention and treatment, the level of glucocorticoid hormone decreases, so that eosinophils gradually increase again in the recovery period of the disease (18, 19). However, there are few studies that point to a specific role for EOS in patients with COVID-19 comorbid with hematological malignancies, and further expansion of the study sample size is needed.

LDH is an intracellular enzyme that plays a crucial role in energy production, primarily synthesized in organs such as the liver, heart, skeletal muscle, kidneys, and lungs. The concentration of LDH in the blood increases in response to tissue damage, cell death, hypoxia (notably due to respiratory failure), hematological and lymphatic disorders, or inflammation affecting the lungs, pericardium, and pancreas (20, 21). Elevated levels of LDH may serve as indicators of tissue hypoxia, cellular damage, or necrosis, conditions that are particularly prevalent in severe COVID-19 patients. Furthermore, existing literature indicates that LDH functions as a serum biomarker associated with poor prognosis in a variety of cancers (22). In some studies, researchers have reported viral involvement in liver function (23–25). An observational study revealed common liver enzyme abnormalities, with increased severity of liver injury, as defined by transaminase or bilirubin abnormalities, being strongly associated with mortality (23). Similarly, our study found higher levels of LDH and ALT in patients who died, indicating their correlation with mortality. Furthermore, our prognosis-related regression analysis identified LDH as an independent risk factor for poor prognosis and in-hospital mortality. Kaplan−Meier survival curves also suggested a higher risk of death in patients with hematological malignancies suffering from COVID-19 in the high-level LDH group. Therefore, high levels of LDH are associated with the worsening course of COVID-19, respiratory failure, and multiple organ dysfunction, making it an important biomarker for assessing prognosis and guiding clinical management in patients with malignant hematological diseases accompanied by COVID-19 infection.

Since the emergence of the COVID-19 pandemic, a notable increase in myocardial injury incidence has been reported and found to be significantly linked to unfavorable prognosis and in-hospital mortality. Peiró et al. discovered that, despite widespread vaccination among patients infected with COVID-19 during subsequent waves, the incidence of myocardial injury in these individuals was comparable to that observed in patients from the initial wave of COVID-19 infections (26, 27). Our study’s statistical analyses revealed a significant elevation of myocardial-associated creatine kinase, troponin T, and NT-proBNP levels in patients in the mortality group. Patients with combined acute myocardial injury exhibited significantly elevated levels of creatine kinase and troponin T. Although the incidence of myocardial injury may vary in different studies, notable elevations in blood cardiac biomarkers independently predict poor in-hospital prognosis and mortality (28, 29). During infection, heightened body metabolism, reduced oxygen supply due to lung lesions, and viral involvement of myocardial tissue can lead to myocardial injury, potentially resulting in adverse outcomes such as malignant arrhythmia, myocardial infarction, and even sudden death (30, 31).

Recent studies have shown a high prevalence of coagulation abnormalities in COVID-19 patients (32). These studies also highlight the impact of hepatic and renal impairment, as well as myocardial dysfunction, on coagulation function. Abudouleh et al. (33) concluded that decreased fibrinolysis is the predominant condition in patients with severe COVID-19. Their study found that elevated coagulation indexes were effective prognostic indicators that could predict patient outcomes at an early stage. Activation of fibrinolysis is recognized as a key mechanism in the progression of respiratory diseases (34). COVID-19 patients experience increased release of fibrinolytic enzymes, which breakdown pulmonary microthrombi. Excessive fibrinolysis increases pulmonary vascular permeability, resulting in reduced fluid and oxygen exchange and disease progression (35). Our study similarly found that patients in the group that succumbed to the disease had elevated levels of FDP, indicating poorer coagulation and hyperfibrinolysis in this group. Coagulation abnormalities and an increased risk of bleeding in the later stages of COVID-19 patients are consistent with the status of disseminated intravascular coagulation (DIC) during sepsis. Furthermore, the administration of large quantities of plasma and fibrinogen during these periods may not always be effective in improving the patient’s condition. The samples in our study comprised patients with preexisting hematological malignancies and coagulation abnormalities. Coagulation abnormalities due to COVID-19 are particularly exacerbated in such patients. Therefore, coagulation abnormalities are also prognostic indicators that require attention during the hospitalization of patients with hematological malignancies who have contracted COVID-19.

## 5 Conclusion

Taken together, our study investigated prognostically relevant risk factors for patients with hematological malignancies and COVID-19. LDH was identified as an independent risk factor for predicting patient prognosis. Clinicians should evaluate not only lung lesions but also the severity of other organ involvement to enhance prognosis assessment. Our study is constrained by its sample size. Future research should aim to increase both the sample size and its diversity while identifying new biomarkers through multicenter collaborations.

## Data Availability

The original contributions presented in the study are included in the article/**Supplementary Material**. Further inquiries can be directed to the corresponding authors.
