# Supplementary material for: Exploration of risk factors and characteristics of COVID-19 infection among patients with hematological malignancies in Suzhou, China: a retrospective study
Source: Front Oncol. 2025 Feb 21;14:1487516. doi: 10.3389/fonc.2024.1487516 (PMC11885061; doi:10.3389/fonc.2024.1487516)
Supplement: Supplementary file 1 [file Table1.docx]

**Supplementary Table 1.** Specific Subtypes of Lymphoma and Leukemia Patients.

| **HM** | **N** |
| --- | --- |
| **Lymphoma** | 23 |
| hodgkin's lymphoma | 1 |
| diffuse large B-cell lymphoma | 13 |
| peripheral T-cell lymphoma | 1 |
| angioimmunoblastic T-cell lymphoma | 1 |
| follicular lymphoma | 6 |
| mantle cell lymphoma | 1 |
| **Leukemia** | 40 |
| acute myeloid leukemia | 27 |
| acute lymphoblastic leukemia | 10 |
| hybrid acute leukemia | 3 |
